# Experiences and expectations in the first trimester of pregnancy: a qualitative study

**DOI:** 10.1111/hex.12572

**Published:** 2017-05-18

**Authors:** Stina Lou, Michal Frumer, Mette M. Schlütter, Olav B. Petersen, Ida Vogel, Camilla P. Nielsen

**Affiliations:** ^1^ DEFACTUM – Public Health & Health Services Research Aarhus Denmark; ^2^ Center for Prenatal Diagnostics Aarhus University Aarhus Denmark; ^3^ Department of Public Health Aarhus University Aarhus Denmark; ^4^ Department of Obstetrics and Gynecology Aarhus University Hospital Aarhus Denmark; ^5^ Department of Clinical Genetics Aarhus University Hospital Aarhus Denmark

**Keywords:** experience, first trimester, pregnancy, prenatal care, qualitative research, ultrasound

## Abstract

**Background:**

A dominant context for pregnant women in the Western world is medical technologies such as ultrasound and screening. It has been argued that such technologies may result in tentative pregnancies, which may be particularly prominent in the first trimester. However, little is known about how women experience early pregnancy.

**Objective:**

To explore the everyday experiences and expectations of first trimester pregnant women in a medicalized context of comprehensive and routine prenatal screening.

**Design:**

Qualitative, semi‐structured interviews analysed using thematic analysis.

**Setting:**

Between May 2015 and January 2016, participants were recruited from two general practices and one obstetric ultrasound unit in Aarhus, Denmark.

**Participants:**

Twenty, first trimester pregnant women (15 primiparae, five multiparae) aged 21‐39 years.

**Results:**

Early pregnancy is often kept secret in the first trimester due to a higher risk of miscarriage. However, the pregnancy is very real in the lives of the pregnant women who make it meaningful through practices of information seeking, listening to the body and anticipating the different milestones in pregnancy. First trimester screening represents one such milestone that is expected to mark a new and more certain phase in the pregnancy. A majority expects to terminate following a prenatal diagnosis, but this does not seem to influence their engagement with the pregnancy.

**Conclusions:**

The pregnant women use medical technologies to mark a milestone in pregnancy but do not expect all concerns to disappear upon a normal screening result. The majority of women acknowledge that pregnancy involves simultaneous feelings of happiness and worry.

## 1 INTRODUCTION

Most women experience pregnancy and childbirth during a lifetime, and as such, it is a common experience. However, for the individual woman, being pregnant is a major and potentially life‐changing event that opens up new possibilities and responsibilities, joys and concerns. Within qualitative research, much attention has been dedicated to the extraordinary biological and social dramas of pregnancy such as infertility, prenatal diagnosis or premature birth. However, within recent years, there has been a growing interest in pregnancy as an everyday experience.^1^, ^2^, ^3^ These studies explore pregnancy through a methodological and theoretical focus on the daily activities through which the pregnancy and future child are made important (or unimportant) in the lives of pregnant women and their partners. This perspective is grounded in an understanding of pregnancy as a socially and culturally embedded process that is constituted through practice. Thus, rather than understanding pregnancy as a biological condition that does something to the woman, pregnancy must be understood as enacted and achieved (or ignored and rejected) through practice. Biologically speaking, pregnancy is an either/or. However, from a social science perspective, pregnancies are made to be through cultural and social practices and the experiences of everyday life.^3^, ^4^ In everyday life, women practice various degrees of acceptance and attachment to the pregnancy and manage and express their pregnancies in numerous ways.

Pregnancies occur within specific social contexts that vary in terms of material and cultural resources and constraints.^5^, ^6^ A dominant context for women in the Western world is the medicalization of pregnancy.^5^, ^7^, ^8^ Several studies have investigated the ever‐expanding medical management and surveillance of pregnancy and pointed to how pregnant women continuously employ and depend on medical technologies—such as screening and ultrasound—to manage their pregnancy.^9^, ^10^, ^11^ Critiques of this development argue that women's embodied knowledge and experience become devalued in favour of biomedical knowledge and evaluation. For example, in her influential analysis of amniocentesis in the United States, Rothman^12^ argues that prenatal surveillance technologies may influence women's experiences of pregnancy and may suspend her attachment to the foetus until biomedical affirmation of its health, resulting in a “tentative pregnancy.” Similarly, other studies have found that pregnant women “withhold the pregnancy announcement”^13^ or try to act “as if they are not pregnant”^14^ in situations where biomedical information questions the health of the foetus.

Denmark offers a good case for investigating how pregnancies are practised in a highly medicalized context. In 2004, Denmark was the first country to implement a comprehensive, national prenatal programme, free of charge, including ultrasound examination in the first and second trimesters as well as first trimester screening (FTS) for Down's syndrome and other chromosomal abnormalities in the foetus. The programme has a very high uptake, and more than 90% of all pregnant women have the FTS performed.^15^ Studies have shown that Danish women generally have a high degree of knowledge of the test concept and a positive attitude towards the FTS.^16^, ^17^, ^18^ Based on this, one could hypothesize that a comprehensive screening programme like the one in Denmark could promote the practice of tentative pregnancies. This may particularly be the case in the first trimester, where bodily symptoms are often subtle and there is no tangible evidence of a future child. However, how women experience early pregnancy and the role played by prenatal screening in these experiences has not been investigated to date.

Thus, the aim of this study was an exploration of the everyday experiences and expectations of first trimester pregnant women in a medicalized context of comprehensive and routine prenatal screening.

## 2 METHODS

An ethnographic methodological approach was used,^19^, ^20^ and qualitative interviews were conducted to explore pregnant women's experience of being in the first trimester.

### 2.1 Study participants

Pregnant women were recruited through two general practitioners and an obstetric ultrasound unit, all located in or close to the city of Aarhus, Denmark. Information pamphlets were distributed to pregnant women coming to the general practitioner (GP) to confirm the pregnancy. In the pamphlet, the aim of the study was explained, and the women were encouraged to contact the researcher. A total of 30 women responded to the invitation. They were contacted by the researcher by phone, given more detailed information about the study and upon verbal consent an interview was arranged. Women who had been to the FTS at the time of contact, or where an interview could not be set up before the FTS, were excluded from the study (n=6). Four women cancelled the interview due to busy schedules, and thus, a total of 20 women were interviewed. Interviews were concurrently transcribed and assessed, and women were included in the study until the researchers estimated that data saturation was met.^21^, ^22^

The interviewed women were between 21 and 39 years of age (average age was 29 years) and in their first trimester at the time of the interview (average gestation was 10 weeks) (Table 1). All women had wanted pregnancies, although some pregnancies were more planned than others. Two women had been in fertility treatment. Five women already had children, and two women had partners with children from previous relationships.

**Table 1:** Participant characteristics

Name	Age	Occupation	Marital status	Gestational age	Children	Early scan (FTS)	Recruited through
Louise	29	Designer of textiles, unemployed	Cohabiting	10+4	–	No	General practitioner (GP)
Cathrine	30	Pedagogic psychologist, unemployed	Cohabiting	11+4	–	No	GP
Maria	25	Student of teaching	Cohabiting	10	–	Yes	Ultrasound unit
Mette	30	Nurse	Cohabiting	12	–	Yes	GP
Camilla	28	Nurse specialist	Cohabiting	12	–	Yes	Ultrasound unit
Mona	22	Student of computer science	Cohabiting	10+5	–	Yes	GP
Julie	34	Physician	Married	11	2	Yes	GP
Sarah	32	Managing secretary	Married	9+2	1	Yes	GP
Christina	35	Occupational therapist	Married	10+3	2	No	GP
Sofie	27	Medical laboratory technician	Cohabiting	6+2	–	No	GP
Tina	39	Nurse specialist	In a relationship	12+0	–	No	GP
Laura	25	Pedagogic assistant	Cohabiting	8+4	–	No	GP
Caroline	26	Student of medicine	Cohabiting	11	–	Yes	GP
Hadiya	21	Unemployed, higher preparatory education course	Married	12	–	No	GP
Anna	26	Nurse	Cohabiting	5+10	1	No	GP
Ingrid	29	Business developer	Married	12+4	–	Yes	Ultrasound unit
Karen	37	Physician	In a relationship	9+4	–	Yes	Ultrasound unit
Charlotte	32	Teacher, occupational therapy	Cohabiting	5+5	–	Yes	GP
Jane	32	Student of business economics	Married	8	1	No	GP
Emma	28	Retail manager	Cohabiting	9	–	Yes	Ultrasound unit

### 2.2 Data collection

The qualitative interviews were performed between May 2015 and January 2016. The interviews were performed by MF and MS, who both have an MA in anthropology and are experienced with ethnographic methodology. The interviews took place in a location of the woman's choice (most often her home) and lasted 20‐40 minutes. All interviews were guided by a semi‐structured interview guide with open‐ended questions (Table 2)^23^, ^24^ developed by all authors in collaboration. The interview themes included as follows: context of the pregnancy; feelings and sensations of being pregnant; experiences with and expectations of prenatal care (eg, ultrasound examinations, midwife appointments); and hopes and thoughts about the pregnancy and future parenthood. After the first two interviews, a few, minor moderations were made to the interview guide to make questions more open and explorative. In all interviews, participants were encouraged to speak freely about their experiences of early pregnancy and bring up topics not covered in the interview guide. All of the interviews were digitally recorded and transcribed verbatim by MF, MS and a student intern.

**Table 2:** Topic guide

Topic	Example of question
The pregnancy in general	Was the pregnancy planned?
	Have you told anyone that you are pregnant? (Why/why not?)
	What is occupying you right now about your pregnancy and being pregnant? (Thoughts, feelings, worries)
Information seeking	Have you sought information about you and your pregnancy? On what? Where, why/why not?
First visit at the doctor	How did you experience the first visit at your doctor?
	What did (s)he tells about prenatal diagnostics?
Early scans	Have you had an early scan? Why? Can you tell about this experience?
The first trimester scan	Have you thought about having the 12‐wk scan?
	What do you expect from the scan? Do you have specific concerns?
	What is a positive result for you?
	Have you thought about getting a high risk assessment? Do you know what your options are then?
	Do you know what you will do in case of a diagnostic result of Down's syndrome?
Life as a parent	Do you at this point think about delivery? What are your thoughts or dreams about life as a parent?
Attitudes towards prenatal diagnostics	What knowledge is important for you in relation to your foetus? (Why this?)
	Is there a limit for what you would want to know about your foetus?
Happy‐worry scale	In general, on a continuum from being worried to being happy in pregnancy, how would you describe yourself?

### 2.3 Data analysis

**Figure 1:**
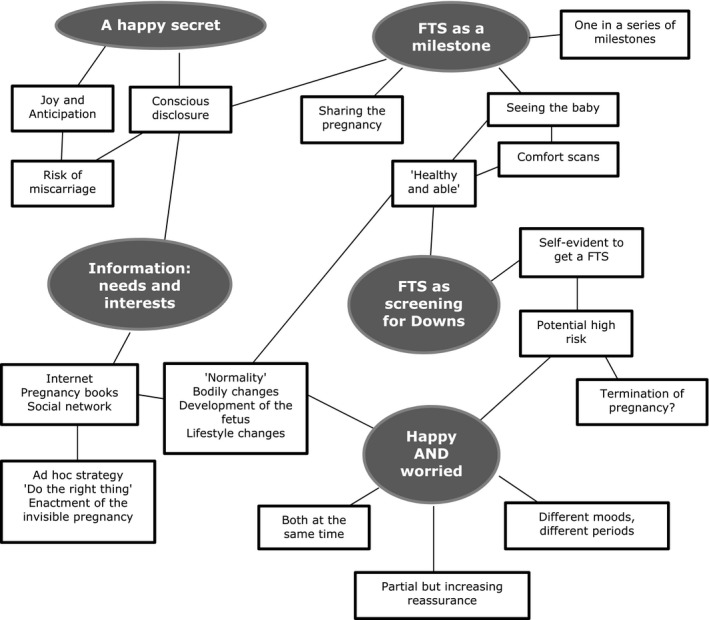
Thematic map

We used thematic analysis^25^ to identify and analyse patterns in the transcribed data. Thematic analysis is a theoretically flexible tool for analysing qualitative data. It is a six‐phase, practical analysis that acknowledges the analytical process as guided by decision and revision rather than simply emerging from the data. The analyses were mainly performed by MF, MS and SL using the following steps. In line with ethnographic methodology, the material was continuously assessed and analytically discussed during the interview process. For the final analysis, all of the material was thoroughly reread to generate initial codes. Two interviews were test‐coded by MF, MS and SL independently, whereupon discrepancies in coding were discussed and the codes refined. Subsequently, all interviews were coded using NVivo 10 software (QSR International, Doncaster, Vic., Australia). The coded material was discussed and sorted into candidate themes in a thematic map (Figure 1), which was discussed by all of the authors. Potential themes were then reviewed, for example to ensure that themes adequately captured the codes and that there was no overlap between themes. Additionally, the themes were investigated in relation to the full data set particularly looking for “negative cases” and disconfirming evidence.^26^ Following this process, the final themes were defined.

### 2.4 Ethical considerations

In Denmark, qualitative research does not require ethical approval from the National Committee on Health Research Ethics (www.nvk.dk). All patients received written and oral information about the study prior to inclusion. It was clearly stated that participation was voluntary and that consent could be withdrawn at any time. Informed consent was confirmed prior to the interview, and all participants have been carefully anonymized in the presentation of the results including through the use of pseudonyms.

## 3 RESULTS

The analysis resulted in the identification of five themes that all played a part in the women's experience and management of early pregnancy: (i) A happy secret, (ii) Information needs and interests, (iii) The FTS as a milestone, (iv) The FTS as screening for Down's syndrome, and (v) Happy and worried.

### 3.1 A happy secret

All women expressed joy and excitement about being pregnant; however, most of them had not fully revealed the pregnancy yet:

It's weird, because usually when you're keeping a secret, then it's a bad thing. It's like: “I don't feel like telling this to anybody.” But this pregnancy has such a strong presence in my mind, and I'm really happy that it does. It's actually something that I'd like to share. It feels weird to have something so special that you can't share yet. I really want to share it. (Ingrid)

A few women had only told their partner, while others had told carefully selected family members or close friends the happy news. Some had felt obliged to inform their workplace because of pregnancy signs such as nausea and fatigue. All anticipated and looked forward to disclosing the pregnancy; however, at this point in the pregnancy, they were consciously selecting and limiting whom they let in on their happy secret.

Being in the first trimester of pregnancy was the major reason for keeping the pregnancy relatively secret. The women all referred to a higher risk of miscarriage in the first trimester as common knowledge. The majority had personal experience with first trimester miscarriages—either themselves or through close friends and relatives. For example, Sofie expressed a level of disbelief and luck that she had not—yet—experienced a miscarriage:

I basically only know people who have had miscarriages… or who have taken years to conceive… and then it just happened so quickly for us. I could hardly believe that it could be so easy. And I just found it really difficult to trust my luck and that it would stay in there. (Sofie)

Thus, even though they felt “very pregnant,” they were unwilling to make the pregnancy fully public and explained this as a kind of “damage control” in case of miscarriage. About half of the women added that the secrecy was also related to the risk of the FTS showing something not developing as expected. Christina explained:

I'm not going to make it public on Facebook and other places before we've been to that scan [FTS] […] I want to get that image, to see that there is actually something in there developing as it should. (Christina)

In sum, concerns about miscarriage and the health of the foetus resulted in the women being very selective in their disclosure of the pregnancy. However, in their minds and in the company of their closest confidants, the pregnancy was very present and very real.

### 3.2 Information needs and interests

Keeping the pregnancy secret meant that some of the women felt alone with their concerns, joys and questions related to the pregnancy. A feeling of having unfulfilled information needs was particularly present before their first GP appointment at app. 8‐10 gestational weeks:

For someone who has never been pregnant before, my head was full of questions. I couldn't really ask anyone in the beginning, because I didn't want to tell our families about it yet, and so I got my information online instead. (Ingrid)

All women had sought information before and after their GP visit, particularly women pregnant with their first child. Some sought advice from mothers, sisters and friends, and some also purchased pregnancy books. All of them used the Internet. The women's information needs and interests were most commonly related to the pregnant body, the developing foetus and lifestyle changes.

First, women were interested in bodily changes and signs related to pregnancy, for example vaginal bleeding or bodily aches and pains. If bodily signs changed or suddenly ceased, women took to the Internet:

I just Google: “Is it normal to”… and then dot dot dot. […] It's a little like, wait, my breasts aren't tender anymore, is that normal? Does it mean that it [the pregnancy] has disappeared? And then I search for that. (Caroline)

The women used the Internet to check the normality of their bodily sensations and eliminate concerns about potential signs of miscarriage.

Second, women sought information about how to calculate the due date and about the weekly development of the foetus:

There is a little description [at sundhed.dk], week for week, what does it look like now, and then I get like a bubbly happy feeling and think that it sounds fun and cute and very weird. (Tina)

And third, the women were interested in different lifestyle concerns, particularly in what changes to make in their everyday lives in order to provide the best possible conditions for the child to be healthy and able:

I have also sought information on pregnancy and working night shifts. And aspirin and pregnancy. […] Just, common everyday life stuff that comes up, really. (Caroline)

I'd been sitting at work eating liquorice and then it suddenly hit me: Wait, that might not be healthy!? And then I immediately Googled it, and ended up at the Danish Health Department's homepage, which had an entire pamphlet about it. (Cathrine)

These quotes show how the women considered their everyday knowledge of night shifts, aspirin and liquorice to be suspended in the new context of the pregnancy. Suddenly, what they knew or what they usually did might be “wrong” or potentially damaging to the development of the foetus. In this rechecking of lifestyle knowledge, the women relied primarily on the Internet. When searching for and assessing the quality of information online, the women generally discerned between factual information and experiential knowledge. For example, sources such as the Danish Health Department's homepage and sundhed.dk (online public health service in Denmark) were frequently mentioned as reliable sources of factual information. However, many women simultaneously sought experiential information and found both relief and entertainment in reading about other women's experiences:

For example, stomach aches. Then I just Google: pregnant, stomach ache. […] Usually I have a very scientific approach, because I'm a nurse, but with this I look at this chat forum, and I think: “Ok, someone has the same as me, ok, calm down.” Those are the kind of situations where I need to hear that someone else has experienced the same and then it's fine. (Laura)

In early pregnancy, the Internet offered a community where women could engage in pregnancy talk, joys and worries with others in lieu of the family and friends to whom the pregnancy had not yet been revealed. A minority of the women consciously avoided these types of blogs and forums to protect themselves from erroneous statements and extreme “horror stories” that might cause more worry than necessary:

It's just all these common people, who are trying to be experts on something, and I really try to stay away from that. (Charlotte)

However, the majority of women stressed the importance of having access to both types of information and inspiration in early pregnancy, and stressed the importance of assessing information with some scepticism and common sense.

In sum, the interviews made apparent that the search for knowledge was driven by an attempt to “do the right thing” or “do all that I can” to give the foetus optimal conditions and preserve the pregnancy in a unique situation where everyday knowledge could not be taken for granted. Searching for information and advice was a pregnancy practice. The interviewed women enacted the early pregnancy through practices such as checking up on liquorice or knowing the foetal developments of their current gestational week.

### 3.3 The FTS as a milestone

All interviewed women mentioned the FTS as the “next milestone” in the pregnancy, and all looked forward to it. First trimester screening was unanimously described as a highly anticipated event that you just “wait and wait and wait” for, in seemingly infinity. Many women spoke enthusiastically about “seeing the baby”:

Well, for me actually it is mostly about the certainty in seeing that there is a baby in my belly and a heart that's beating… well… that is what it's mostly about for me. (Emma)

In a situation of no visible signs of pregnancy and often elusive bodily symptoms, the FTS promised visual certainty and a biomedical confirmation of pregnancy. Additionally, the women looked forward to sharing the experience with the father:

Frederik, my boyfriend, he finds it a bit difficult to relate to this whole thing. That there actually is something […] as he says, it will be entirely different the day we go to the 12‐week scan. Where he also can see that there is something. (Anna)

Notably, eleven women out of 20 had already had one or more ultrasounds performed, either at the hospital or in private clinics. The two women in fertility treatment had scans to confirm the pregnancies. Some women had received an early scan by “accident” as they had been estimated to be further in their pregnancy than they actually were, and the others paid to have an extra scan early in the pregnancy. These women referred to their extra scans as “a reassuring scan” or “a comfort scan.” These women actively used ultrasound as a tool for eliminating worries about a viable foetus and continued pregnancy. However, even for women who had already seen the foetus, the FTS was reported to be special: it not only confirms the pregnancy and the health of the foetus, but also marks the transition into the second trimester and to the fully revealed pregnancy.

After the FTS, the women expected and anticipated to publicly share the news of the pregnancy. Some had made arrangements for meeting parents or friends the following weekend and had selected situations in which they expected to share the news. During interviews, it became apparent that the women perceived not only the FTS as a milestone, but the pregnancy as a series of milestones to be accomplished, from the stripes on the pregnancy test to the birth of a healthy child:

I actually kind of look forward to the FTS. To get this pregnancy started, if you can say that […] because it's things like these you really associate with being pregnant, I think. When you hear about it from others saying “Yes, now we've been to the first scan.” It's like one of these milestones, which are kind of associated with all this about being pregnant. (Maria)

In sum, the FTS represents a personal and social milestone; a step towards future parenthood and a procedure that adds social value to the pregnancy and the pregnant woman.

### 3.4 The FTS as screening for Down's syndrome

The women appreciated that the FTS was optional, and that some women may decline to participate. However, the interviewed women all expressed it as a self‐evident choice to attend. The women referred to participation in FTS as “a no‐brainer,” an “obvious” or a “natural” decision:

It's really not something we have discussed. It's just something that I presumed we both wanted to do. (Louise)

All of the women knew that the FTS would produce a “risk” or “probability” of Down's syndrome in the foetus, and that invasive diagnostics would be available in the case of a high‐risk screening result. When asked whether they had considered how to respond to an abnormal screening result and diagnostic test, only one woman explicitly stated that she, due to religious convictions, would not terminate the pregnancy. Seven women explicitly stated that they would terminate:

If we find out that the child has Down's syndrome, then we will terminate. No doubt. Because I don't think… well, I don't think it's fair to…. Well, first of all it puts a lot of strain on the parents to have such a child, but also the child… it's not going to be a very easy life, is it? My cousin is handicapped and she is so dependent. I just don't think that I'd want to consciously create a life like hers. There are so few things she can do, she needs help and constant surveillance and I just don't want to purposely create that situation for someone. (Jane)

The women emphasized the strain of having a handicapped child, the burden on siblings and the worries and concerns of having a child that would not be able to care for itself even in adulthood. Some added that they had not experienced difficulties in getting pregnant and, thus, were confident that they could get pregnant again (with a healthy foetus).

Six of the interviewed women expected to terminate the pregnancy (for the same reasons as mentioned above), but recognized that that they might feel differently in the actual situation:

My boyfriend was very quick to say that if it has Down's then he wouldn't want it. And that is probably also my first thought, but I don't know… when you are in the situation […] if you feel differently when you've actually seen that “the little one” in there already has arms and legs and all that. If you sort of relate to the child? I don't know. (Sofie)

These women emphasized that it is hard to predict how one will react in such an emotional situation and, thus, made room for potential reconsideration and decisional conflict.

Finally, six women had not thought about a potential abnormal result or discussed it with their partner:

No, actually we haven't. If something comes up, then I think we will have to take it from there. (Julie)

These women explicitly stated that they did not want to worry about unpleasant scenarios that would most likely not happen. They deliberately postponed a decision until relevant, based on the hope and expectation that the foetus was healthy and the pregnancy was normal.

Thus, the majority expected to terminate the pregnancy in the case of foetal diagnosis. However, they considered it a very small risk and stated that, overall, it did not influence their excitement and joy about the pregnancy, although several women mentioned that they would probably get nervous just prior to the FTS.

### 3.5 Happy and worried

To illuminate how the women understood the uncertainties of early pregnancy, we asked each woman to position herself on a happy/worried continuum with a carefree and happy pregnant woman at one end and a worried and concerned pregnant woman at the other end of the continuum. Interestingly, most women related happiness and worries and described themselves as “being more happy than worried,” “on the happy end, even though I have my worries” or “rather non‐worried.” The women described their feelings as a mixture of different moods during different periods and seemed to have no difficulties in being both at the same time, for instance, Mette reflected on our question and suggested we modify it:

Well, I don't know, because I am very, very, very happily pregnant. But of course I also have some worries […] but I think that my happiness clearly overshadows my worries […]. Well actually I think that it is rather difficult to describe with a scale. Perhaps it would be better if you could bend the scale in the shape of a U, then I could be very worried AND very happy at the same time. (Mette)

A few women described themselves as more worried than happy, but hoping to eliminate some worries as the pregnancy developed and more milestones were achieved. However, they expressed an understanding that certainty is always partial as other concerns about the continued health of the child would undoubtedly emerge throughout the pregnancy. Among the interviewed women, there was an overall approach to the pregnancy that nothing is certain and that there are no guarantees:

I just really want the FTS to be over. Not because lots of things can't happen later, but… it's just nice to know that you can cross Down's syndrome and those kinds of things off the list. […] I realize, it's sort of a false feeling of safety. It's a little like walking away from there thinking: “Great, there's nothing wrong,” but it's still quite possible that something is anyway. (Camilla)

It's not like I'm thinking that just because everything looks fine at the FTS, that I will be completely convinced that everything else will also go well. (Mette)

The women did not expect the FTS to guarantee a healthy child and did not expect to leave their worries completely behind them following a normal FTS result. Rather, the continued intermingling of joy and worry, of uncertainty and reassurance was understood as integral to the pregnancy experience as a whole. Being worried and being happy are both ways of practising and engaging with the early pregnancy.

## 4 DISCUSSION

The women in this study were generally excited about their pregnancy. The results show that the higher risk of miscarriage in the first trimester was a main reason for not fully disclosing the pregnancy until after the end of the first trimester or the FTS. In this new context of pregnancy, the women felt that previous everyday knowledge could not necessarily be trusted, and thus, seeking information and advice was a common practice. The women discerned between factual information and experiential advice and used them for different ends. As one of several milestones in the pregnancy, the FTS was anticipated to mark a new and more certain phase in the pregnancy. The majority were inclined to terminate the pregnancy in case of diagnosis of Down's syndrome, whereas the rest had deliberately postponed any decision making. Overall, the results show how the early pregnancy involves a continuous intermingling of joy and worry, and that medicalizing technologies such as ultrasound and expert knowledge are incorporated into these experiences without defining them in any simple way.^27^

The findings add to the growing body of knowledge on normal pregnancy and demonstrate the ways in which early pregnancy comes into being in the women's lives by the ways they think, speak and act. Seeking information or eagerly waiting for the FTS is a practice that makes pregnancy real, important and meaningful in the women's lives—even before ultrasound confirmation. Thus, our findings add a more nuanced perspective to the concept of the tentative pregnancy. While many of the women in the study do withhold the pregnancy from public announcement, they do not ignore the pregnancy or passively await medical confirmation. Rather, they engage in micro practices—searching for information, listening to the body—that establish the pregnancy and give it a place in their lives.

The findings demonstrate how everyday knowledge is suspended in the new context of pregnancy: former knowledge no longer necessarily applies. Pregnant women are surrounded by an overabundance of expert and lay advice on how they should regulate their bodies and protect the pregnancy.^3^, ^4^ This causes some authors to argue that pregnancy and childbirth have become like medical diseases.^5^, ^8^ However, the women in our study do not act as if they are diseased. Rather, our findings resonate with other studies^28^, ^29^, ^30^ documenting the complex ways in which this surveillance and regulation of pregnancy are actively and creatively reproduced by the women, as these practices are simultaneously ways of making the pregnancy real and being a responsible mother to the foetus.

Most of the interviewed women expected to terminate the pregnancy in case of a Down's syndrome diagnosis; however, this willingness to terminate did not appear to impact their excitement about being pregnant. This can be understood as an ad hoc approach to decision making in pregnancy that has also been found in several other studies.^31^, ^32^, ^33^ For example, it has been well documented that women do not seek information about the biomedical complexities and potentially very difficult decision making that may follow from a high‐risk screening result, an abnormal finding or a prenatal diagnosis. Instead, women rely on the health professionals to inform, guide and counsel them should a difficult situation arise.^34^, ^35^, ^36^

We speculate that this selective delegation of knowledge and responsibilities to health professionals and to (unlikely) future scenarios allow women to engage in the early pregnancy despite the highly medicalized, Danish context and their pronounced decision to terminate in case of diagnosis.

Our findings add an interesting perspective on the dynamics of excitement and worry in early pregnancy. Several qualitative and quantitative studies have investigated levels of worry and anxiety in pregnancy as well as the impact of prenatal surveillance such as ultrasound and screening on worry and anxiety.^37^ However, there is a tendency to assume that worry or increased anxiety is always abnormal or undesirable, and that worry and joy are mutually exclusive. However, first of all, it has been argued that reasonable levels of worry may be appropriate in situations where there is a great deal at stake, and important decisions must be made.^38^, ^39^ The pregnant women in the present study do have their worries, as they hope to keep the pregnancy and they wish to make good choices that support the health of the foetus. Second, our results demonstrate that being worried does not necessarily exclude simultaneous feelings of joy and excitement in pregnancy.

### 4.1 Methodological limitations

When assessing the results and conclusion of this study, a number of methodological considerations must be taken into account. First, the women were informed about the study by health professionals, which may result in selection bias. We do not know whether the professionals refrained from approaching women they considered vulnerable or unfit for the study. Second, the sample is self‐selected, and we have no information on non‐participation. The sample consists of pregnant women with time, resources and interest in contacting a researcher. This may explain why the sample is biased towards higher education. Consequently, the women in this sample may be more inclined to use information seeking as a means to practicing the early pregnancy, and thus, we may overestimate the importance of information. Third, none of the women in the present study had serious hereditary disease or a genetic risk profile, which may cause considerably more worry and uncertainty in early pregnancy than reported by the women in our sample.

Finally, in interviews, we rely on what the interviewees tell us. There may be practices that women do not disclose because they deem them irrelevant, embarrassing, forget them or find them difficult to put into words. Future studies should include observations of pregnant women's everyday lives to get a denser and more nuanced understanding of the practices of early pregnancy.

## 5 CONCLUSIONS

This research has found that early pregnancy may be withheld from public announcement in the first trimester due to a higher risk of miscarriage. However, the pregnancy is very real in the lives of the pregnant women, and they make it meaningful through practices of information seeking, listening to the body and anticipating the different milestones in pregnancy. The FTS represents one such milestone after which the women expect to be less worried. A majority expect to terminate following a prenatal diagnosis, but this does not seem to influence their engagement with the pregnancy. In the medicalized, Danish context, the majority categorize themselves as “happy”. However, they do acknowledge that pregnancy involves simultaneous feelings of happiness and worry.

## CONFLICT OF INTEREST

None declared.
